# Study of Mo (VI) removal from aqueous solution: application of different mathematical models to continuous biosorption data

**DOI:** 10.1186/1735-2746-10-14

**Published:** 2013-01-25

**Authors:** Fatemeh Kafshgari, Ali Reza Keshtkar, Mohammad Ali Mousavian

**Affiliations:** 1 Department of Chemical Engineering, Faculty of Engineering, University of Tehran, Tehran, Iran; 2 Nuclear Fuel Cycle School, Nuclear Science and Technology Research Institute, Atomic Energy Organization of Iran, Tehran, Iran

**Keywords:** Biosorption, Molybdenum, Packed bed column, Marin algae, *Cystoseria indica*

## Abstract

Molybdenum (VI) biosorption process was investigated by marine algae *Cystoseria indica* pretreated with 0.1 M CaCl_2_ solution in a packed bed column. The biosorbent was characterized by FTIR, BET and SEM analyses. The results showed that Mo (VI) ions should be chelated with the hydroxyl, carboxyl and amine groups of the biomass. The effects of inlet metal concentration and flow rate on biosorption process were investigated and the experimental breakthrough curves were obtained. Results showed that the maximum biosorption capacity of Ca-pretreated *C. indica* for Mo (VI) was found to be 18.32 mg/g at optimum flow rate of (1.4 mL/min). The controlled-rate step shifted from external to internal mass transfer limitations, as the flow rate increased. Also, it was observed that the breakthrough and exhaustion time decreased from 17.14 hr to 9.05 hr and from 0.006 h to 0.002 hr respectively, with the increase of flow rate from 0.7 to 2.1 ML/min. The increase in the initial concentration of Mo (VI) solution from 30 to 95 ml min^-1^ increases the adsorption capacity from 18.32 to 30.19 mg/g and decreases the percentage of Mo (VI) removal from 61 to 38%. Also, the treated volume was the greatest (1.42 L) at the lowest inlet concentration. Column data obtained under different conditions were described using the Thomas, Yoon and Nelson, Yan and Belter models. The breakthrough curve predictions by Belter model were found to be very satisfactory.

## Introduction

Continuous discharge of industrial, domestic and agricultural wastes in rivers and lakes causes deposit of pollutants in sediments. Such pollutants include heavy metals, which endanger public health after being incorporated in food chain. Heavy metals are present in different types of industrial effluents, being responsible for environmental pollution [1]. Incidence of heavy metal accumulation in fish, oysters, mussels, sediments and other components of aquatic ecosystems have been reported from all over the world [2,3].

Many types of yeast, fungi, algae, bacteria and some aquatic plants have been reported to have the capacity to concentrate metals from dilute aqueous solutions and to accumulate them inside the cell structure [4-6].

The potential application of microorganisms as biosorbent for the removal of heavy metals has been recognized as an alternative to the existing conventional methods for detoxification of industrial wastewaters [7,8].

Molybdenum is an essential trace element for both plants and animals [9]. Molybdenum deficiency has often been reported but in large concentrations, molybdenum may be toxic as it leads to secondary copper deficiency [10]. Therefore, the removal of this ion from wastewater and groundwater is of significant importance from an environmental point of view.

One of the heavy metal removal technologies called biosorption uses non-living microorganisms in treating wastewater containing heavy metals [11,12].

The role of various microorganisms by biosorption in the removal and recovery of heavy metal(s) has been well reviewed and documented [13-18]. It is particularly the cell wall of certain algae, fungi and bacteria which is found to be responsible for the phenomenon of biosorption [19].

Among these, the brown algal biomass has been shown to be highly effective, reliable, and predictable in the removal of heavy metals from the aqueous solutions [20-22].

The packed bed adsorption has a number of advantages: it is simple to operate, attains a high yield and it can be easily scaled up from a laboratory-scale procedure. The stages in the separation protocol can also be automated and high degrees of purification can often be achieved in a single step process [23].

In our previous study [24], we showed that the pretreatment of *Cystoseria indica* by 0.1 molar CaCl_2_ solutions improved the uptake capacity of uranium more than 30%. In the present work, the molybdenum (VI) biosorption by the Ca-pretreated brown algae (*Cystoseria indica)* was investigated in a packed bed column. The effect of design parameters, such as flow rate and influent concentration on molybdenum (VI) biosorption were examined. For a proper design of a biosorption column, an accurate prediction of the breakthrough curve is needed. Therefore, the experimental results obtained from the continuous flow packed bed column were fitted to the Thomas, Yoon and Nelson, Yan and Belter models.

## Materials and methods

### Preparation of biosorbent

The brown alga, *Cystoseria indica* was obtained from The Persian Gulf on the coast of Qeshm, It was thoroughly washed with distilled water and sun-dried on the beach and in an oven at 50°C overnight. The dried biomass was ground in a laboratory blender and sorted using the standard test sieves. The batch of biomass with particle size of 1.0-1.25 mm was selected for subsequent Ca-pretreatment. Ca-pretreatment of the biomass was carried out as follows: a sample of 10 g of biomass was treated with 0.1 M CaCl_2_ solution (1000 mL) for 12 hours under slow stirring. Then the biomass was washed with distilled water. Finally, the biosorbent was again dried at 70°C for 8 h.

### Chemicals

The standard solutions of Mo (VI) were prepared by diluting 1000 mg/L Mo (VI) solution by dissolving analytical grade sodium molybdate (Na_2_MoO_4_.2H_2_O) in water. Diluted solutions were prepared at room temperature in distilled and deionized water. The pH of influent solutions was adjusted with a pH meter (Metrohm, Model 780) by using 0.1 M HCl and/or 0.1 M NaOH.

### Continuous flow column system

All of the experiments were performed in a packed-bed column at room temperature (25 ± 2°C) and pH = 2. A preliminary analysis of the acid mine water from a low grade uranium ore leaching factory indicates that the concentration of Mo (VI) ions is less than 100 mg/L; therefore in this paper, the effect of Mo (VI) ion concentration on the column performance was investigated in the range of 30 to 95 mg/L. The column was a simple glass tube with inner diameter of 1.5 cm and length of 10 cm. Two plastic sieves both with pore size of 0.5 mm were installed at the top and bottom of this column. The experiments were conducted by pumping a metal solution in up-flow mode through the packed-bed column with a peristaltic pump (Watson Marlow Pumps, Model 205 U). A weight of 1 g of the dry biosorbent (equal to 4 cm bed height) was packed within the column.

### Measurements and methods

The functional groups of the biosorbent were determined by a Fourier Transform Inferred Spectrometer (Vector22-Bruker Company, Germany) in the range of 400-4000/cm wave number. The morphological analyses of the biosorbent were characterized using a scanning electron microscope (SEM, JEOL JSM-6380). The specific surface area and pore volume were estimated using Brunauer–Emmett–Teller (BET) method. The analysis was carried out through an adsorption of N_2_ gas (gas effluent temperature of 300°C and bath temperature of 77.3°C). Micro pore volume was calculated from the volume of nitrogen adsorbed at relative pressure (P/P_o_) of 0.9893. Liquid samples were analyzed for residual metal ion concentration by an inductively coupled plasma spectroscopy (ICP, Varian, Model Liberty 150 AX Turbo). Analytical wavelength was set at 202.031 nm for Mo (VI) ions.

### Analysis and mathematical modeling of breakthrough curves

The design and optimization of packed-bed columns are difficult to carry out a priori without a quantitative modeling approach. From the perspective of process modeling, the dynamic behavior of a packed bed column is described in terms of the effluent concentration-time profile, i.e. the breakthrough curve. Dividing the metal mass (m_ad_ the quantity of metal retained in the column (mg)) by the sorbent mass (M) leads to the maximum uptake capacity of the biosorbent [25,26]. The breakthrough time, *t*_b_, is said to be occurred when the effluent metal concentration reaches 5% of the influent value (hr) and bed exhaustion time, *t*_e_, is assumed to be occurred when the effluent metal concentration is equal to 95% of the influent value. Also, the effluent volume (V_e_) that is the volume of metal solution passed into the column (L) can be calculated as follows [25,27]:

(1) $$V_{e}=0.06\times Q\times t_{e}$$

where *t*_e_ is time (h) and Q is the volumetric flow rate (mL/min). Total amount metal sent to column m_total_ (mg) can be calculated as follows [25]:

(2) $$m_{\mathrm{total}}=0.06\times C_{0}\times Q\times t_{e}$$

Total metal removal percent with respect to flow volume can be calculated as follows [25]:

(3) $$\mathrm{Total}\;\mathrm{metal}\;\mathrm{removal}\;\left(\%\right)=\frac{m_{\mathrm{ad}}}{m_{\mathrm{total}}}\times 100$$

Successful design of a continuous packed bed column generally requires prediction of concentration-time profiles for the effluents (i.e. breakthrough curves). The maximum uptake capacity of a biosorbent is also needed. There are several mathematical models in the literature, which have been used to represent the dynamics of the packed bed column. Thomas, Yoon- Nelson, Yan, and Belter models are among them, which can be written respectively as below [28-31]:

(4) $$\frac{C}{C_{0}}=\frac{1}{1+\exp\left(\frac{K_{\mathrm{Th}}q_{0}M}{0.0001*Q}-K_{\mathrm{Th}}C_{0}t\right)}$$

(5) $$\frac{C}{C_{0}}=\frac{1}{1+\exp\left(K_{\mathrm{YN}}\tau -K_{\mathrm{YN}}t\right)}$$

(6) $$\frac{C}{C_{0}}=1-\frac{1}{1+{\left(\left(\frac{0.001\times C_{0}\times Q}{q_{0}\times M}\right)\times t\right)}^{a}}$$

(7) $$\frac{C}{C_{0}}=\frac{1}{2}\left(1+\mathrm{erf}\left[\frac{t-t_{0}}{\sqrt{2}\sigma \;t_{0}}\right]\right)$$

where K_Th_ is the Thomas rate constant (l/min. mg), q_0_ is the maximum uptake capacity (mg/g). The kinetic coefficient k_Th_ and the adsorption capacity of the bed q_0_Th can be determined from a plot of ln ((C_0_/C) −1) against *t* at a given flow rate.

M is the amount of adsorbent in the column (g), C_0_ is the influent metal ion concentration (mg L^-1^), C is the effluent metal ion concentration (mg/L), Q is the flow rate, K_YN_ is the Yoon-Nelson rate constant (1/min), τ is the time required for 50% adsorbate breakthrough (min). The parameters k_YN_ and τ may be determined from a plot of ln (C/(C_0_ − C)) versus sampling time (t). t is the flow time (min) and “a” is the constant of the Yan model.

Also, erf (x) is the error function of x, t_0_he temporal parameter (min) which indicates time needed for the outlet metal concentration to be the half of the one inlet metal concentration and σ is the standard deviation of the linear part of the breakthrough curve in the Belter model.

## Results

### Adsorption mechanism of Mo (VI) on the C. indica brown algae

**Figure 1:**
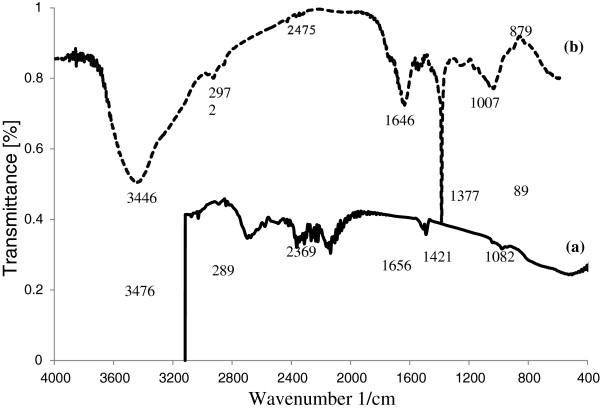
FTIR spectrum of (a) unloaded and (b) Mo (VI)-loaded Ca-pretreated C. indica biomass.

Fourier Transform Infrared (FTIR) spectroscopy has proven and accepted to be a powerful tool for the study of biological samples [32]. Therefore, the algal samples were studied using FTIR technique. FTIR spectra of Mo (VI) unloaded and loaded forms of the biosorbent in the range of 400–4000/cm wave number were taken and presented in Figure 1. As shown in Figure 1, the unloaded biomass displays a number of absorption peaks, indicating the complex nature of the biomass examined.

**Figure 2:**
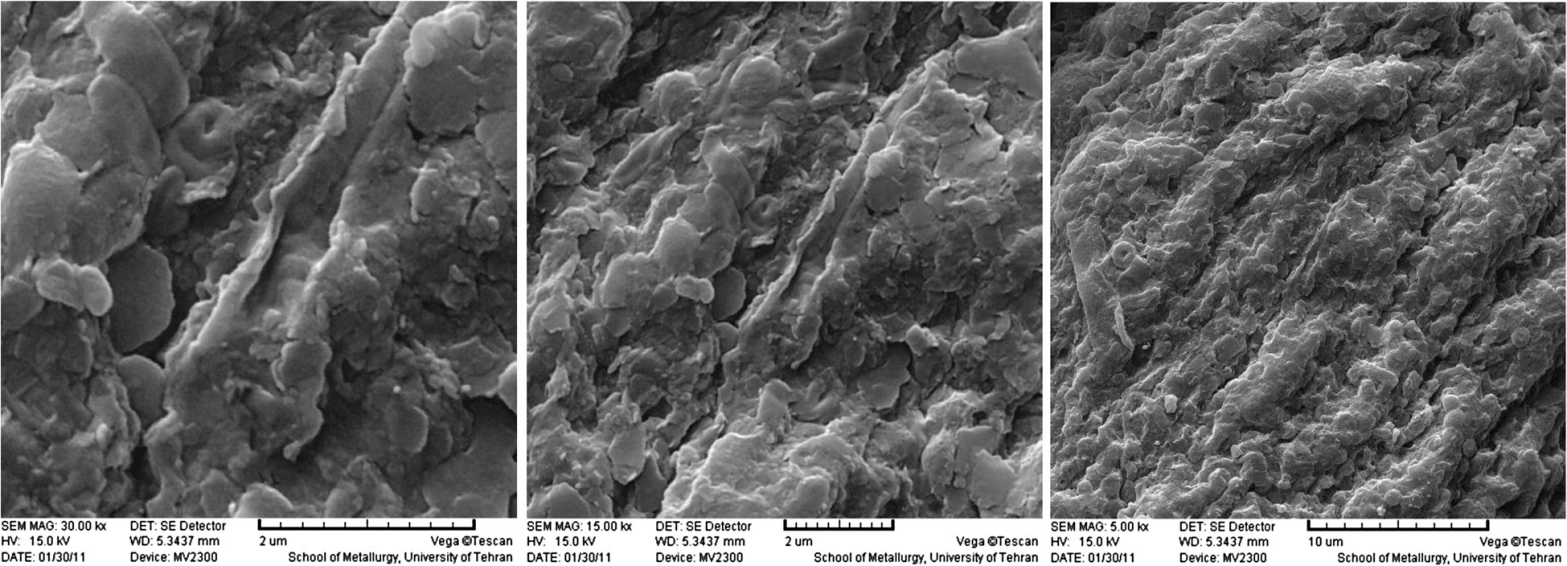
**SEM image of Ca-pertreated *C. indica *biomass at different magnifications.**

Also, some physical characteristics such as, Surface area, pore volume, and pore diameter of the Mo-loaded Ca-pretreated *C. indica* and unloaded Ca-pretreated *C. indica* biomass were determined on the basis of the Brunauer, Emmet and Teller (BET) method and the results were shown in Table 1. By the scanning electron microscopy (SEM), the biomass surface SEM image at different magnifications was obtained and is shown in Figure 2.

**Table 1:** **Physical properties of unloaded and Mo (VI)-loaded Ca-pretreated *C. indica *biomass**

**Property**	**Unloaded biomass**	**Mo(VI)-loaded biomass**
Moisture (%)	0.890	0.740
Density (g/cm)	1.112	1.241
Pore volume (cm^-3^ g^-1^)	5.259E-3	2.700E-2
Pore size (nm)	2.871	1.184
Porosity (%)	0.585	3.350
Specific area (m^2^/g)	36.710	31.660

### The effect of flow rate

**Figure 3:**
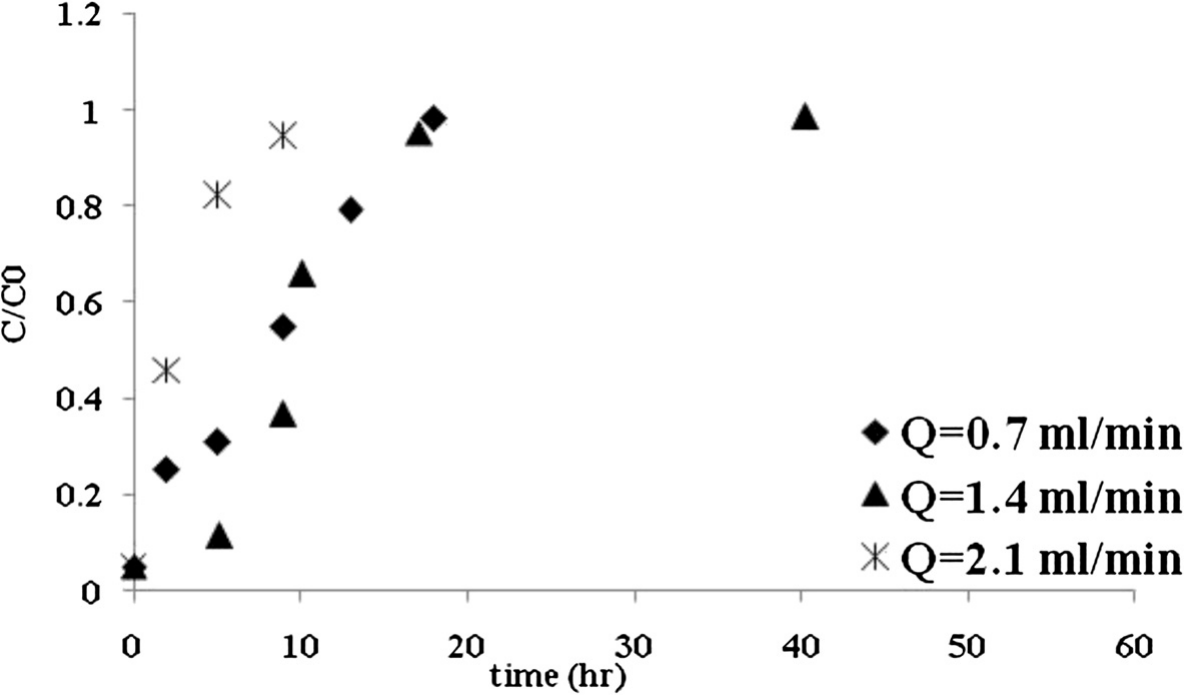
**Breakthrough curves for Mo (VI) biosorption onto *C. indica *at different flow rates.**

The effect of flow rate on Mo (VI) biosorption by *C. indica* was studied by varying the flow rate from 0.7 to 2.1 mL/min, while the bed height and initial Mo (VI) concentration were held constant at 4 cm and 30 mg/L, respectively. Figure 3 shows the breakthrough profile of Mo (VI) biosorption at different flow rates and the results of the breakthrough curve analysis are given in Table 2. Results showed that the breakthrough and exhaustion time decrease with the increase of the flow rate from 0.06 to 0.02 h and from 17.14 to 9.05 h respectively. Also, in this range, the metal uptake capacity was strongly influenced by increasing flow rate from 0.7 to 1.4 mL/min, so that, its value for molybdenum increased from 10.32 to 18.32 mg/g. As can be seen from Table 2 when the flow rate was increased from 1.4 to 2.1 mL/min^,^ the molybdenum removal capacity was decreased from 18.32 to 11.7 mg/g.

**Table 2:** **Column data and parameters obtained at 30 mg/L inlet metal ion concentration and different flow rates for Mo (VI) biosorption onto Ca-pretreated *C. indica *biomass**

**Q (mL/min)**	**t**_**e**_**(h)**	**V**_**eff**_**at t**_**e**_**(l)**	**t**_**b**_**(h)**	**Uptake capacity (mg/g)**	**τ**_**exp**_**(h)**	**Ad (%)**
0.7	17.14	0.72	0.06	10.32	8.16	47
1.4	17.01	1.42	0.05	18.32	5.51	61
2.1	9.05	1.14	0.02	11.7	2.34	35

### The effect of influent concentration

**Figure 4:**
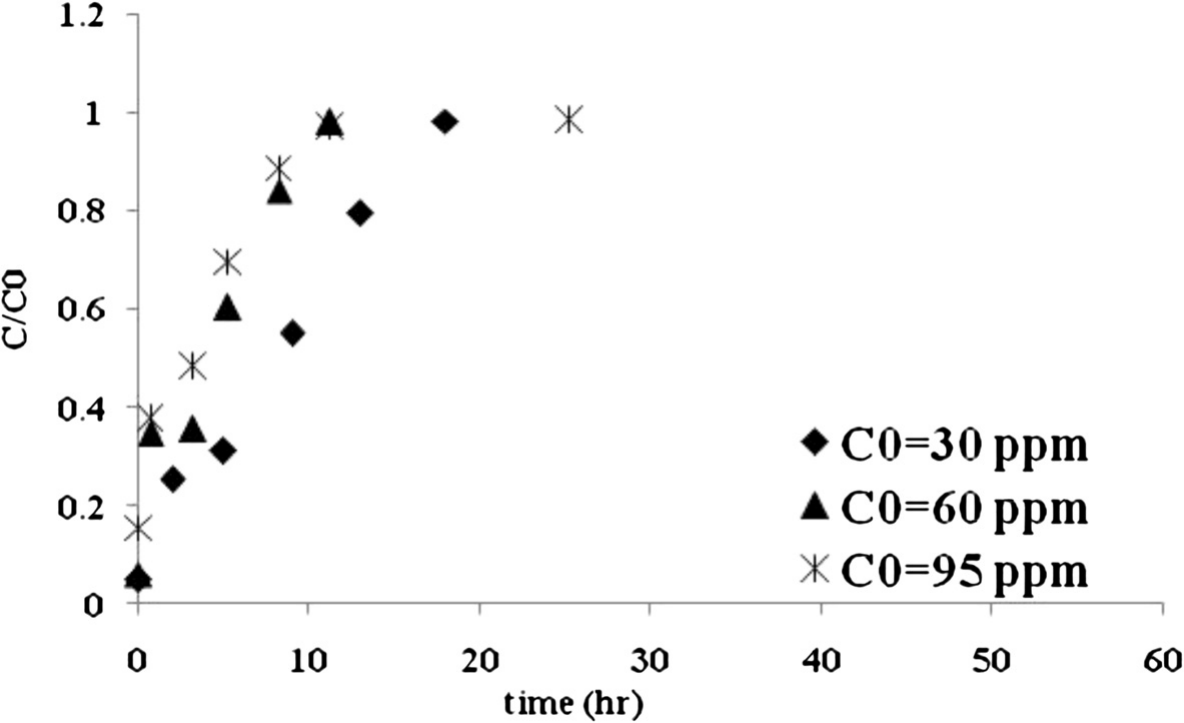
**Breakthrough curves for Mo (VI) biosorption onto *C. indica *at different influent concentrations.**

The change in the influent metal ion concentration has a significant effect on breakthrough curve. The breakthrough curves obtained by changing inlet Mo (VI) concentration from 30 to 95 mg/L at 4 cm bed height and 1.4 mL/min flow rate are shown in Figure 4. The increase in the initial concentration of Mo (VI) solution increases the adsorption capacity from 18.32 to 30.19 mg/g and decreases the percentage of Mo (VI) removal from 61 to 38% (Table 3). Also, the breakthrough time and exhaustion time decreased with an increase in initial concentration as shown in Table 3, the treated volume was the greatest (1.42 L) at the lowest inlet concentration.

**Table 3:** **Column data and parameters obtained at 1.4 mL/min and different inlet metal ion concentrations for Mo (VI) biosorption onto Ca-pretreated *C. indica *biomass**

**C**_**0**_**(mg/L)**	**t**_**e**_**(h)**	**V**_**eff**_**at t**_**e**_**(l)**	**t**_**b**_**(h)**	**Uptake capacity (mg/g)**	**τ**_**exp**_**(h)**	**Ad (%)**
30	17.010	1.420	0.05	18.320	5.51	61
60	10.570	0.887	0.04	20.650	4.42	42
95	10.500	0.882	0.03	30.190	3.39	38

### Model of column data

The design and optimization of a fixed-bed sorption column involves the employment of mathematical models, which must be used to describe and predict the experimental breakthrough curves, in order to make the scale up of the process possible. Because of this, the experimental breakthrough curves have been fitted to each one of the models mentioned previously with the aim to describe the behavior of the column for biosorption of Mo (VI) onto *C. indica* and to determine the corresponding kinetics parameters. The experimental breakthrough curves have been fitted to the models through the non-linear regression, using the MATLAB software.

**Figure 5:**
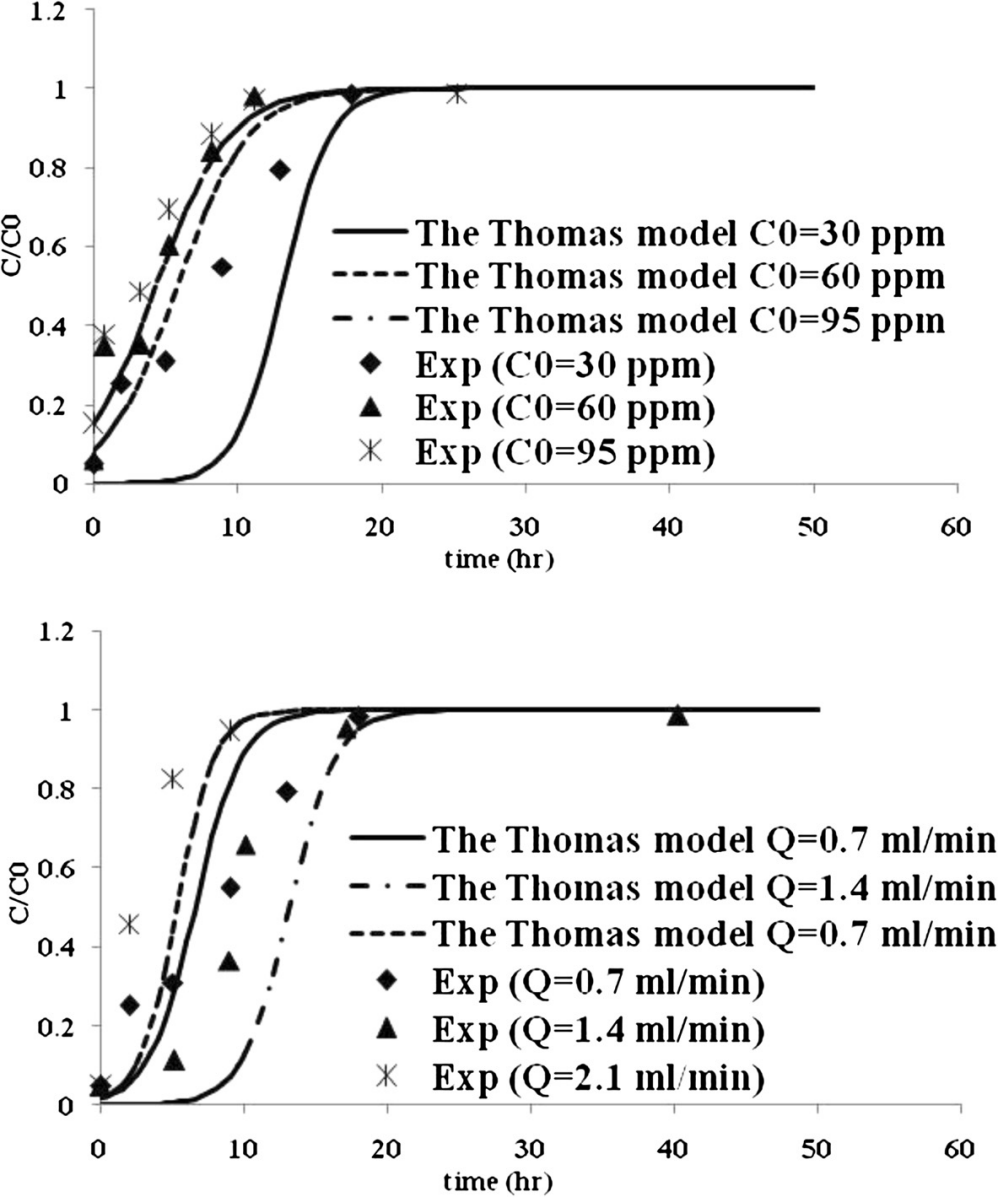
**Comparison of the experimental and model fit breakthrough curves for Mo (VI) biosorption by *C. indica *at different flow rates and influent concentrations according to the Thomas model.**

**Figure 6:**
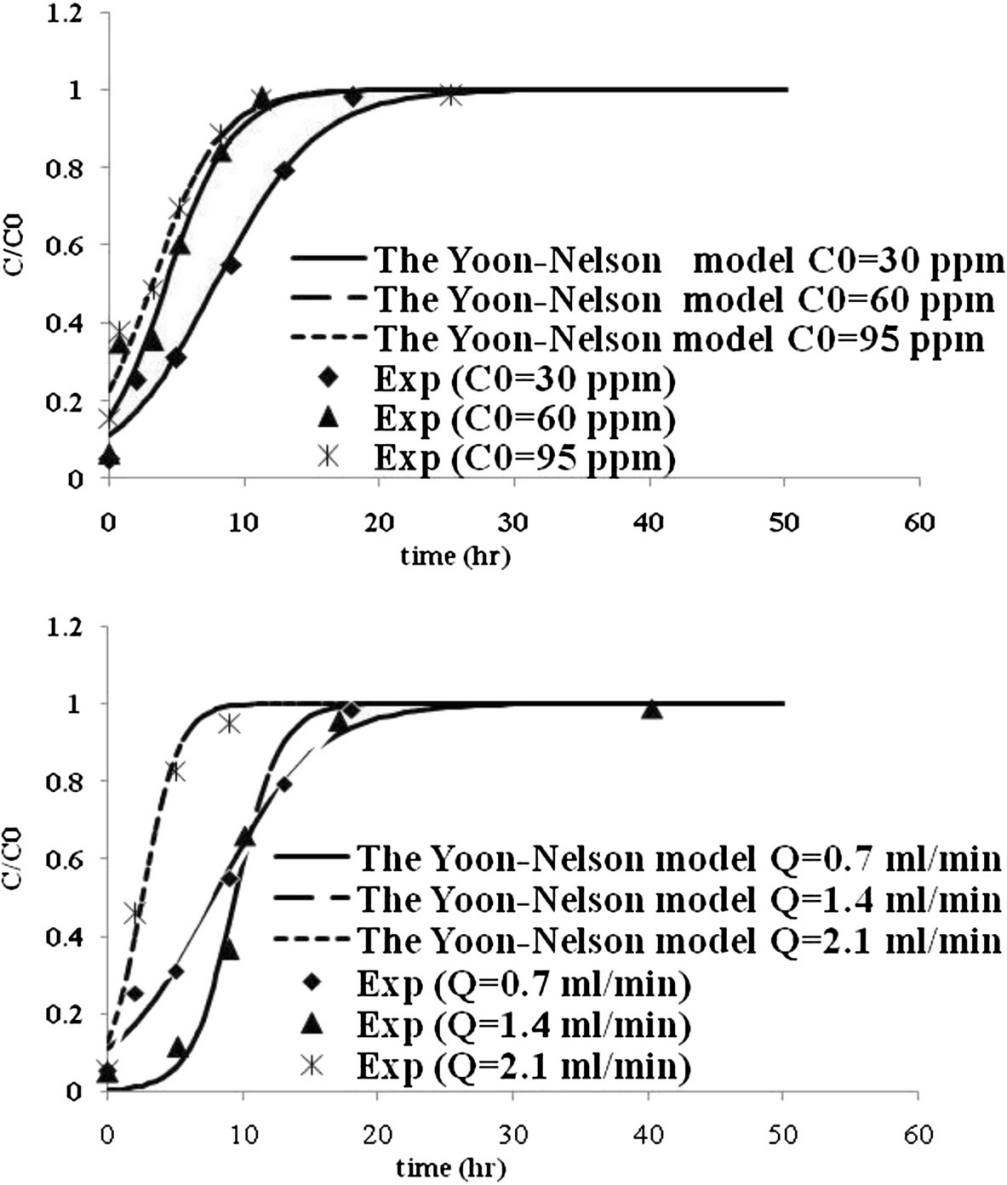
**Comparison of the experimental and model fit breakthrough curves for Mo (VI) biosorption by *C. indica *at different flow rates and influent concentrations according to the Yoon and Nelson model.**

**Figure 7:**
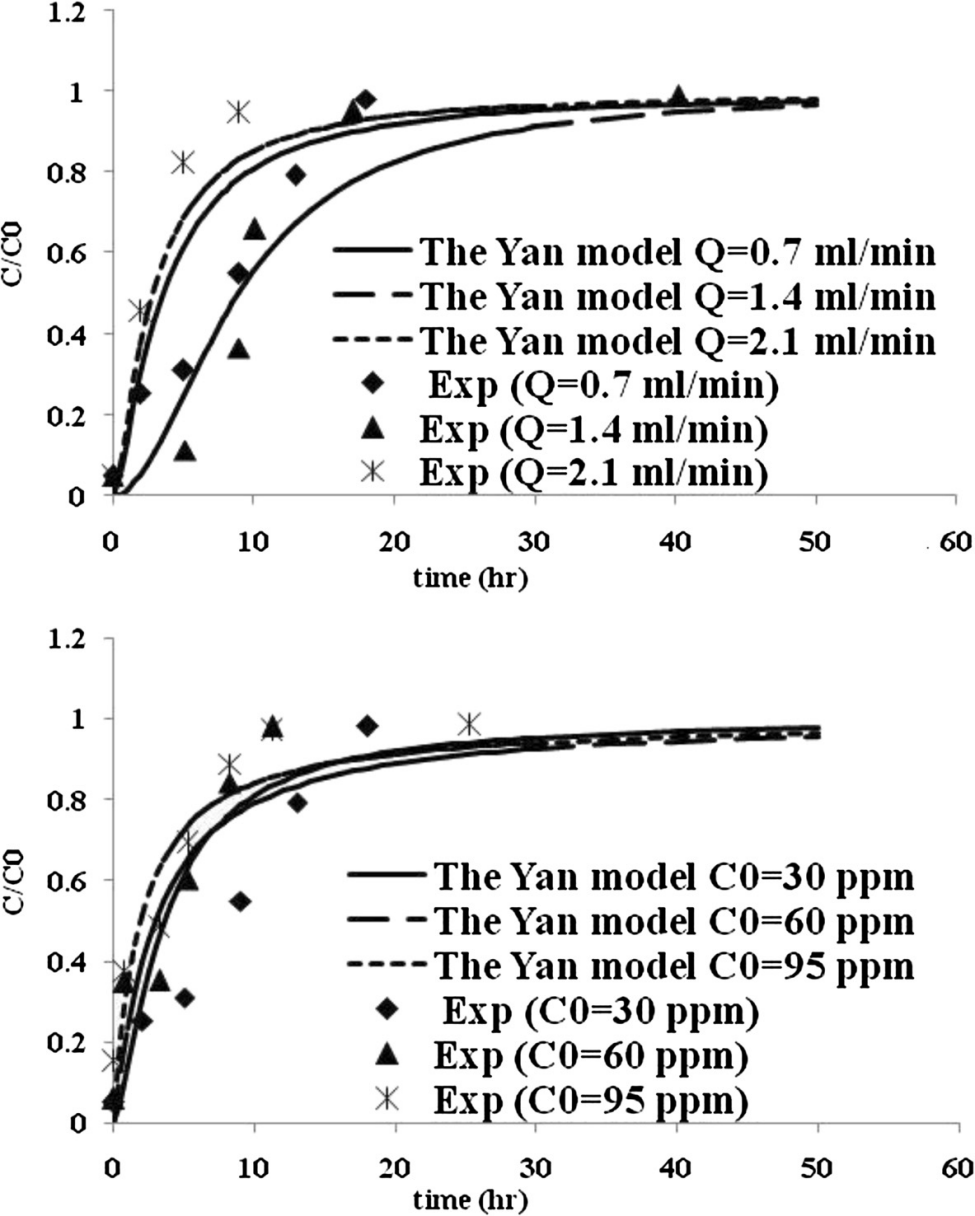
**Comparison of the experimental and model fit breakthrough curves for Mo (VI) biosorption by *C. indica *at different flow rates and influent concentrations according to the Yan model.**

**Figure 8:**
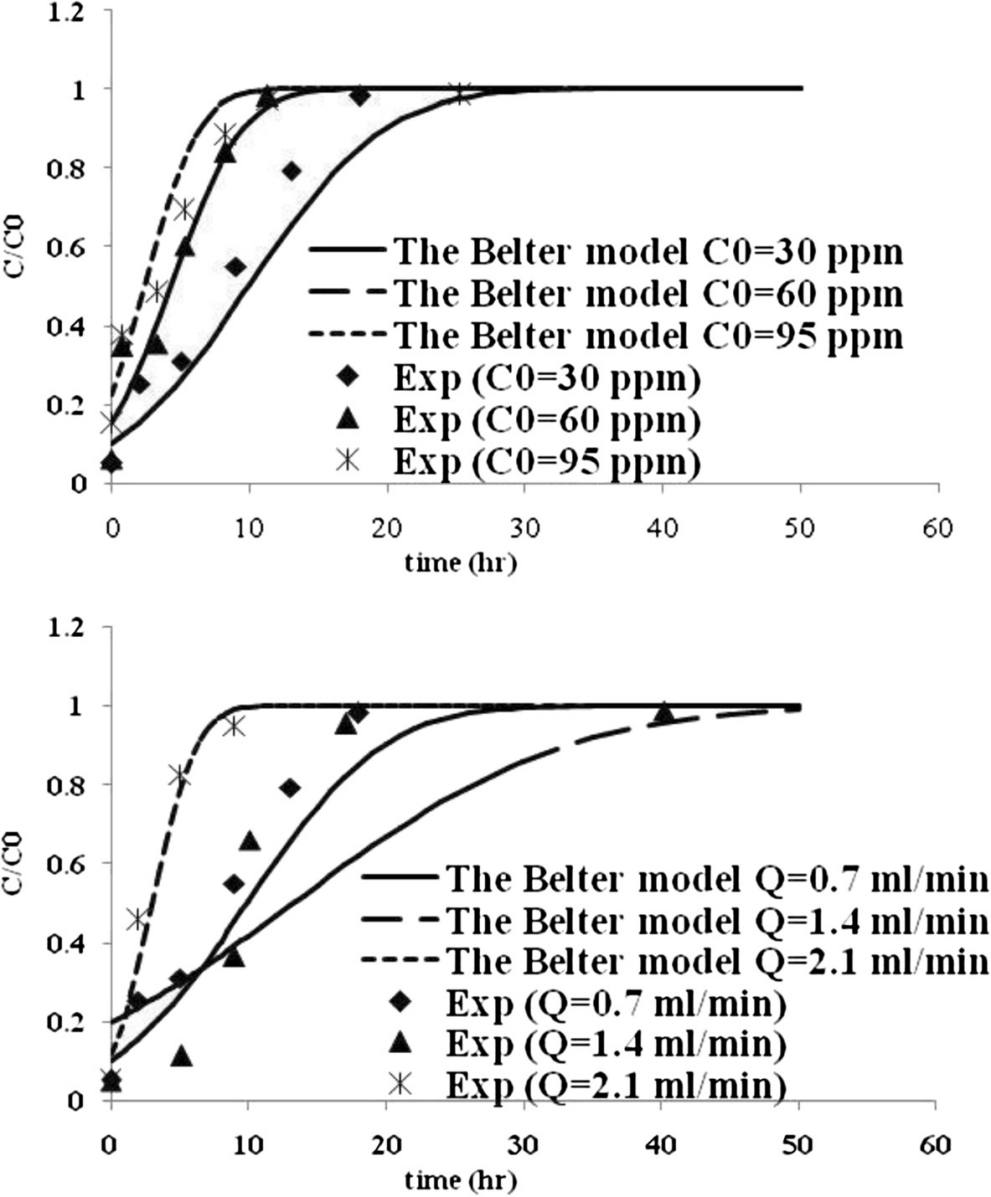
**Comparison of the experimental and model fit breakthrough curves for Mo (VI) biosorption by *C. indica *at different flow rates and influent concentrations according to the Belter model.**

Table 3 lists the model parameters of Thomas, Yoon and Nelson, Yan and Belter models with R^2^ values. Figures 5, 6, 7 and 8 show the experimental points and predicted breakthrough curves in different conditions according to the Thomas, Yoon and Nelson, Yan and Belter models, respectively.

As can be seen, these models (except the Yan model) were found to be most suitable to represent the kinetics of biosorption of Mo (VI) in a packed bed of *C. indica*. But the Belter model was better.

## Discussion

### Adsorption mechanism of Mo (VI) on the C. indica brown algae

Molybdenum exhibits multiple valences of +3, +4, +5 and +6. The stable valence of +6 is important, not only for extraction but also for chemical adsorption mechanism. Predominating ionic species of Mo (VI), such as MoO_2_^2+,^ MoO_4_^2-^, HMoO_4_^-^ and neutral species H_2_MoO_4_ are reported to be present in a molybdenum aqueous solution [33]. Such species play a vital role to understand the biosorption mechanism of molybdenum onto *C. indica* biomass. Brown algae contain 20-40% of alginic acid on a dry weight basis. Alginic acid, a linear polysaccharide, is a main cell wall constituent of brown algae. Numerous chemical groups have been proposed to be responsible of biosorption metal binding by algae (hydroxyl, carboxyl, sulfhydryl, sulfonate, etc.); their importance for metal uptake depends on factors such as the quantity of sites, its accessibility, chemical state or affinity between site and metal [20]. The confirmation of the presence of these groups has been achieved by FTIR analysis. When the pH of solution decreases the hydroxyl groups on the cell walls of biomass is protonated and the surface of biomass is positively charged that causes an increase in electrostatic adsorption of anionic species of molybdenum. A similar phenomenon has also been reported for Mo (VI) adsorption onto persimmon residua [34].

In order to further explain the adsorption mechanism of Mo (VI), FTIR spectroscopies of biomass before and after molybdenum adsorption were investigated. The FTIR spectra of unloaded biomass showed broad bands at 3476/cm that represent bounded hydroxyl (−OH) and amine (−NH_2_) groups. The bands observed at 2899/cm^/^could are assigned to the aliphatic C-H group. The peak around at 2369/cm^/^represent stretching vibrations of -NH_2_^+^, -NH^+^ and –NH groups of the biomass. The adsorption bands at 1656/cm and 1421/cm were attributed to stretching vibration of carboxyl group (−C = O). The peak around at 1082/cm were due to the C–O stretching of alcohols and carboxylic acids. The bands at 893 cm^-1^ can be attributed to the aromatic C-H bending vibrations. Also, the strong band around 1100-1000/cm are due to the C-OH bond, which is a characteristic peak for polysaccharides [35]. After Mo (VI) biosorption, the stretching vibration bands of hydroxyl and amine groups were shifted to 3446/cm for Mo (VI)-loaded biomass. The vibration bands of aliphatic C-H group were shifted to 2972/cm. The stretching vibration bands of carboxyl groups were shifted to 1646 and 1377/cm. The bands assigned to C–O stretching and aromatic C-H bending vibrations were also shifted to 1007 and 879/cm, respectively. These changes observed in the spectrum indicated the possible involvement in Mo (VI) biosorption process of those functional groups on the surface of the biomass. In addition, the disappearance of the band at 2369/cm indicated that the chemical interactions between the protonated amine group of the biomass and molybdenum ions was mainly involved in the biosorption.

As can be seen from Table 2 the Mo (VI) biosorption process onto *C. indica* biomass led to change of density from 1.112 to 1.241 g cm^−3^. By comparison of biomass density with water density, we can observe that the biomass has a high degree of settle ability. The implication is that the biomass would be suitable for the continuous flow systems. Also, specific area decreased from 36.71 to 31.66 m^2^/g and porosity increased from 0.585 to 3.35%. These observations indicated that the chemical adsorption of Mo (VI) ions changes the cell wall structure of biomass.

### The effect of flow rate

As can be seen from Table 2, the breakthrough curves became steeper and the breakthrough and exhaustion time decreased with the increasing flow rate. This behavior may be due to insufficient residence time of the solute in the column, which causes the Mo (VI) solution to leave the column before equilibrium occurs. On the other hand, the results showed that in the range of low flow rates (high residence times), the overall rate of Mo (VI) ions removal in the packed column was controlled by external mass transfer limitations. At low flow rates (from 0.7 to 1.4 mL/min), the metal uptake was strongly influenced by increasing the flow rate, so that, its value increased from 10.32 to 18.32 mg/g. In addition, the results showed that in the range of higher flow rates (lower residence times), the overall rate of metal sorption by *C. indica* biomass was controlled by diffusion limitations of the solute into the pores of sorbent. When the flow rate increased from 1.4 to 2.1 mL/min, the liquid residence time in the column decreased, resulting in a lesser biosorption of metal ions, and hence, the metal uptake decreased from 18.32 to 11.7 mg/g. Similar observation was reported by Vijayaraghavan *et al.*[36].

The sensitivity of the biosorption to the liquid flow rate can be explained by the fact that the liquid residence time in the column is critical for the biosorption process. When the process is subjected to external mass transfer control, a higher flow rate decreases the liquid film resistance and when the process is subjected to intraparticle mass transfer control, a slower flow rate favors the sorption.

### The effect of influent concentration

One of the parameters that strongly affect the metal removal capacity as well as the general position of the breakthrough curve is the initial metal ion concentration. According to the obtained results the uptake capacity increases as the inlet Mo (VI) concentration due to the increasing of driving force for mass transfer. This result is in agreement with that reported by Vijayaraghavan *et al.*[36,37], Han *et al.*[38] and Malkoc *et al.*[39] studies. On the other hand, the percentage of Mo (VI) removal decreases as the influent concentration of Mo (VI) increases because, when the column gets close to saturation, the quantity of metal biosorbed is very low in comparison to the quantity of metal that passes through the column. A similar trend was also observed for Cr (III) biosorption by olive stone [40].

As influent concentration increased, the breakthrough curve became steeper and shifted towards the origin for Mo (VI) ions as the binding sites became more quickly saturated in the system at higher initial concentrations.

Also, as shown in Table 3, the treated volume was the greatest at the lowest inlet concentration since the lower concentration gradient caused a slower transport due to a decreased mass transfer coefficient.

### Model of column data

Thomas model has been applied to the breakthrough curve prediction. The calculated q_0_ value is maximized in flow rate of 1.4 mL/min and 30 mg/g inlet metal concentration, which agrees with the one obtained by the experimental data (Table 4, Figure 5).

**Table 4:** **Thomas, Yoon-Nelson, Belter and Yan model parameters at different flow rates and inlet metal ion concentrations for Mo (VI) biosorption onto Ca-pretreated *C. indica *biomass**

**Experimental conditions**	**Q (mL/min)**	**0.7**	**1.4**	**2.1**	**1.4**	**1.4**
	**C**_**0**_**(mg/L)**	**30**	**30**	**30**	**60**	**95**
Thomas model	q_0_	9.910	16.125	9.571	21.082	23.678
	K_Th_	0.008	0.020	0.025	0.007	0.004
	R^2^	0.979	0.984	0.973	0.941	0.975
Yoon-Nelson model	τ (h)	7.865	6.399	2.532	4.183	3.132
	K_YN_	0.266	0.611	0.756	0.402	0.391
	R^2^	0.979	0.984	0.973	0.941	0.975
Belter model	σ	0.794	1.188	0.852	0.987	1.341
	t_0_ (h)	9.895	13.25	3.020	4.235	2.349
	R^2^	0.982	0.990	0.973	0.943	0.976
Yan model	q_0_	4.545	15.4	10.49	13.7	14.27
	a	1.418	1.903	1.409	1.087	0.996
	R^2^	0.956	0.946	0.946	0.833	0.881

According to the obtained results in Table 4, the sensitivity of the Thomas model constants with the changes of the liquid flow rates and the existence of a maximum in the q_0_ values as the flow rate increases show that the controlled-rate step shifts from external to internal mass transfer limitations and the model is able to predict it. Also, the kinetics constant, K_Th_, tends to increase as the flow rate increases and decrease as the influent metal concentration increases.

For the Yoon and Nelson model, the calculated τ values are quite close to those found experimentally, which indicates that the parameters of the model are similar to those obtained in the experiments (Tables 2, 3, 4 and Figure 6). On the other hand, the time necessary to reach 50% of the retention, τ, significantly decreases when the flow rate and influent metal concentration increase, because the saturation of the column occurs more rapidly.

The equation proposed by Yan was not able to fit the experiment breakthrough curves in a non-linear analysis (Figure 7). The parameters obtained by non-linear fit of the data, q_0_, and ‘a’ along with correlation coefficients is listed in Table 4. As can be seen from Table 4, the Yan model does not adequately reproduce the experimental data, obtaining R^2^ values less than 0.956. Also, the q_0_ values predicted by this model are lower than those found from the experimental data, especially in higher inlet molybdenum concentrations, which once again indicates that it is not possible to reproduce the results using this model.

The Belter model parameters t_0.5_ and σ for the biosorption of Mo (VI) on Ca-pretreated *C. indica* are found by fitting Eq. (7) to the experimental data (Table 4). Breakthrough curves calculated using these fit values for t_0.5_ and σ are shown in Figure 8, in comparison with the experimental data. The model showed a good fit to most of the obtained column data. However, the model parameters of the Belter show no apparent dependence on the flow rate and inlet metal ion concentration variables. It must be taken into account that these empirical model parameters lack any physical significance and are only valid under the same conditions applied to obtain the breakthrough curves, because they can change with variables such as the flow rate, inlet metal ion concentration, column dimensions, or packed algae. Nevertheless, this shortcoming is also present when the simplified mechanistic models are used. To summarize, it can be said that the Belter model is able to describe the complete breakthrough curve, suggesting that the features of the dynamics of sorption column have been taken into account by the mathematical form of the model equation.

## Conclusions

This study indicated that the Ca-pretreated C. indica biomass could be used as an efficient biosorption bed in the fixed-bed columns for the removal of MO (VI) ion bearing aqueous solutions because, C. indica is an inexpensive, easily available biomaterial with high adsorption capacity for MO (VI) ion. The effect of concentration and flow rate on biosorption process was investigated and the experimental breakthrough curves were obtained. Also, it was observed that there was an optimum flow rate of 1.4 ml min^-1^ for metal ion in the column.

Thomas, Yoon-Nelson, Belter and Yan models were applied to the experimental data obtained from the biosorption of MO (VI) ion onto C. indica. Among these models, the Belter model appeared to describe the experimental results better.

Thus, Cystoseira indica can be used in the fixed bed column as a potential biosorbent for treatment of Mo (VI) polluted aqueous solutions.

## Competing interests

The authors declare that their competing interests are biosorption study, mathematical modeling, Thermodynamics, Kinetics.

## Authors’ contributions

FK: The biosorption studies have been done, the related experiment have been and the reports have been supplied. ARK: The biosorption studies have been done and the reports have been supplied. MAM: The reports have been supplied. All authors read and approved the final manuscript.
